# In vitro characterization of a small molecule PD-1 inhibitor that targets the PD-l/PD-L1 interaction

**DOI:** 10.1038/s41598-021-03590-4

**Published:** 2022-01-07

**Authors:** Chih-Hao Lu, Wei-Min Chung, Chun-Hao Tsai, Ju-Chien Cheng, Kai-Cheng Hsu, Huey-En Tzeng

**Affiliations:** 1 The Ph.D. Program of Biotechnology and Biomedical Industry, China Medical University, Taichung, 40403 Taiwan; 2 Department of Medical Laboratory Science and Biotechnology, China Medical University, Taichung, 40403 Taiwan; 3 Graduate Institute of Biomedical Sciences, China Medical University, Taichung, 40403 Taiwan; 4 Department of Orthopedics, China Medical University Hospital, Taichung, 40403 Taiwan; 5 Department of Orthopedics, School of Medicine, China Medical University, Taichung, 40403 Taiwan; 6 Department of Sports Medicine, College of Health Care, China Medical University, Taichung, 40403 Taiwan; 7 Graduate Institute of Cancer Biology and Drug Discovery, Taipei Medical University, Taipei, 11031 Taiwan; 8 Ph.D. Program for Cancer Molecular Biology and Drug Discovery, Taipei Medical University, Taipei, 11031 Taiwan; 9 Division of Hematology/Oncology, Department of Medicine, Taipei Medical University Hospital, Taipei, 11031 Taiwan; 10 School of Medicine, Taipei Medical University, Taipei, 11031 Taiwan; 11 Department of Medical Research, Taichung Veterans General Hospital, Taichung, 40705 Taiwan; 12 Division Of Hematology & Oncology, Taichung Veterans General Hospital, Taichung, 40705 Taiwan

**Keywords:** Drug discovery, Immunology

## Abstract

Targeting the programmed cell death protein 1/programmed cell death ligand 1 (PD-1/PD-L1) axis with monoclonal antibodies (mAbs) represents a crucial breakthrough in anticancer therapy, but mAbs are limited by their poor oral bioavailability, adverse events in multiple organ systems, and primary, adaptive, and acquired resistance, amongst other issues. More recently, the advent of small molecule inhibitors that target the PD-1/PD-L1 axis have shown promising cellular inhibitory activity and the potential to counteract the disadvantages of mAbs. In this study, structure-based virtual screening identified small molecule inhibitors that effectively inhibited the PD-1/PD-L1 interaction. Six of those small molecule inhibitors were applied to cell-based experiments targeting PD-1: CH-1, CH-2, CH-3, CH-4, CH-5, and CH-6. Of all 6, CH-4 displayed the lowest cytotoxicity and strongest inhibitory activity towards the PD-1/PD-L1 interaction. The experiments revealed that CH-4 inhibited the interaction of soluble form PD-L1 (sPD-L1) with PD-1 surface protein expressed by KG-1 cells. Investigations into CH-4 analogs revealed that CH-4.7 effectively blocked the PD-1/sPD-L1 interaction, but sustained the secretion of interleukin-2 and interferon-γ by Jurkat cells. Our experiments revealed a novel small molecule inhibitor that blocks the interaction of PD-1/sPD-L1 and potentially offers an alternative PD-1 target for immune checkpoint therapy.

## Introduction

By negatively regulating the immune system, programmed cell death protein 1 (PD-1) helps to prevent the development of autoimmune disease^1,2^. When this immune checkpoint binds to its ligand, programmed cell death ligand 1 (PD-L1), the resulting interaction inhibits immune responses, stimulates the release of cytokines and triggers cytotoxic reactions^3^, suppressing T cell functions^4^. Under normal physiological conditions, PD-1 is expressed by a variety of immune cells including T lymphocytes^1^. PD-L1 expression allows different types of tumor cells (e.g., melanoma, colorectal and renal cells) to escape immune system onslaughts^5–7^. The advent of immune checkpoint therapy, specifically designed to block the PD-1/PD-L1 interaction by either directly targeting tumor cells or non-specifically revamping the immune system, relies on PD-L1-induced downregulation of the immune mechanism and the resulting suppression of cancer cell growth^8,9^.

Anti-PD-1/PD-L1 monoclonal antibodies represent a crucial breakthrough in anticancer therapies^9,10^. Many countries, including the United States, Japan and Europe, have approved several anti-PD-1/PD-L1 therapies for treating multiple tumors, including head and neck cancer, melanoma, lung cancer, and ovarian cancer^11–13^. The benefits of such treatment are not limited to cancer, with evidence suggesting that regulating the immune response via the PD-1/PD-L1 interaction can also apply to infectious diseases such as tuberculosis, human immunodeficiency virus (HIV) infection, hepatitis and malaria^14–17^.

Several monoclonal antibodies against PD-1/PD-L1 have been introduced into the clinic and have been reported on extensively^18–20^. For instance, PD-1 antibodies pembrolizumab and nivolumab^21–23^ have proven efficacy in various cancers, such as bladder cancer and Hodgkin’s lymphoma^21,24,25^. However, current immunotherapeutic strategies that use PD-1 or PD-L1 antibodies are associated with important disadvantages, including the following: inflammation and detrimental effects on the skin, gastrointestinal, hepatic and endocrine systems^24^; over two-thirds (~ 70%) of patients administered such treatment either fail to respond or gain only short-term benefits and experience disease recurrence within a short time of completing treatment^26^; treatment-related increases in immune system activity can induce undesirable events such as myocarditis, vasculitis, heart failure, dermatitis, endocrine dysfunction, and even death^27,28^; cancer immunotherapies have been associated with primary, adaptive, and acquired resistance^29^; long half-lives and persistent side effects^30^. Researchers have acknowledged the vital importance of identifying new immune-related oncology molecular targets and small molecule drugs that will enable immunotherapies to treat a larger range of tumors and more diverse patient populations, with fewer side effects and less treatment resistance^30^.

Small molecule inhibitors (SMIs) have attracted much interest in cancer research for their potential applications in immunotherapy. SMIs offer several advantages over large-molecule inhibitors including monoclonal antibodies such as greater cell permeability, organ specificity, shorter biological half-lives, cheaper production costs, and the possibility for oral administration^31,32^. Moreover, compared with conventional therapies, SMIs are associated with a greater number of possible targets and routes for suppressing oncogene expression^2,31,32^. Other advantages of SMIs over existing monoclonal antibodies include higher stability and better tumor penetrance^33^. Preclinical studies have demonstrated that SMIs exhibit promising tumor suppression by interrupting the PD-1/PD-L1 interaction^34,35^.

However, using SMIs to target the PD-1/PD-L1 interaction has been challenging due to the relatively flat and hydrophobic surfaces where these proteins interact, which makes the physical placement of inhibitors on those surfaces extremely difficult^31,36^. In our quest to improve SMI immunotherapy, we applied protein docking analysis to examine the structure and aspects of the PD-1/PD-L1 interaction in our attempt to identify similar or identical structures to the PD-1/PD-L1 binding epitope. Predicted compounds were tested for their inhibitory capacities in in vitro experiments. In this paper, we have identified a novel SMI that specifically targets PD-1 and effectively blocks the PD-1/PD-L1 interaction. This SMI shows minimal cytotoxicity in different T cell populations and maintains their secretion of interleukin (IL)-2 and interferon gamma (IFN-γ).

## Results

### Anchors of the receptor-binding site

**Figure 1:**
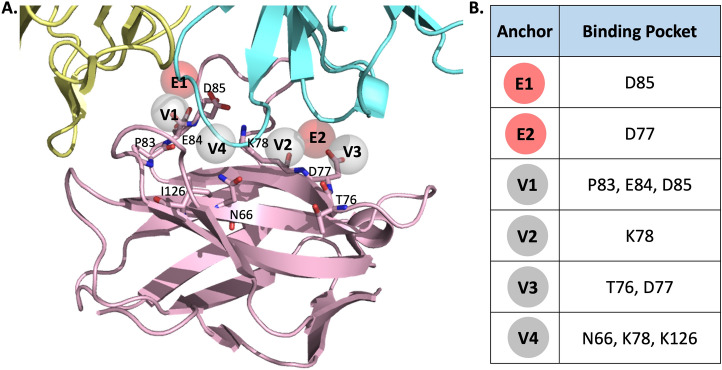
Anchors obtained from SiMMap. (**A**) The structure of the human PD-1 in complex with pembrolizumab Fv (5B8C) is depicted as a cartoon and six anchors as transparent spheres. PD-1 is colored in pink, while the light and heavy chains of pembrolizumab are colored in yellow and cyan, respectively. Red E1 and E2 represent the electrostatic force; gray V1, V2, V3 and V4 represent van der Waals forces. The corresponding binding pockets (residues) are represented as sticks. (**B**) The table presents binding pockets for each anchor.

Six pivotal anchors (E1, E2, V1, V2, V3 and V4) were generated by SiMMap in the PD-1 receptor-binding site. The phosphate moieties in the E1 and E2 anchors prefer to interact with acidic residues, D85 and D77, respectively. The four anchors of the van der Waals interactions have a high tendency to bind to an aromatic and heterocyclic moiety: V1 interacts with one hydrophobic residue (P83) and two acidic residues (E84 and D85); V2 interacts with one basic residue (K78); V3 interacts with one nucleophilic residue (T76) and one acidic residue (D77); V4 interacts with one amide residue (N66), one basic residue (K78) and one hydrophobic residue (I126). Our 3-dimensional (3-D) model is shown in Fig. 1. The top 20 compounds ranked by SiMMap scores were selected as potential candidates and requested from the NCI (Supplementary Table S1). The six available compounds were quantified for cytotoxicity and inhibitory capacities, using an in vitro cell cytotoxicity assay and a cell-based PD-1/PD-L1 inhibitor screening assay.

### In vitro characterization of the inhibitory capacities of CH compounds

Protein docking analysis identified six potential SMIs targeting PD-1: CH-1, CH-2, CH-3, CH-4, CH-5, and CH-6. To evaluate their capacity to inhibit the PD-1/PD-L1 interaction, we used a cell-based PD-1/PD-L1 inhibitor screening approach consisting of a bioluminescent cell-based assay containing two main components; genetically manipulated effector cells (growth-arrested reporter Jurkat cells) and engineered PD-L1/TCR-overexpressed HEK293 cells (target cells). In this assay, PD-1-expressed effector cells were genetically manipulated with the nuclear factor of activated T-cells (*NFAT*)-associated luciferase reporter gene. We used HEK293 cells for transient overexpression of PD-L1 and an engineered TCR activator as target cells. When the two cells were co-cultivated, the PD-1/PD-L1 interaction prevented TCR activation and suppressed *NFAT*-induced luciferase activity. Inhibition of the PD-1/PD-L1 interaction activated TCR signaling in effector cells and upregulated luminescence intensity.

**Figure 2:**
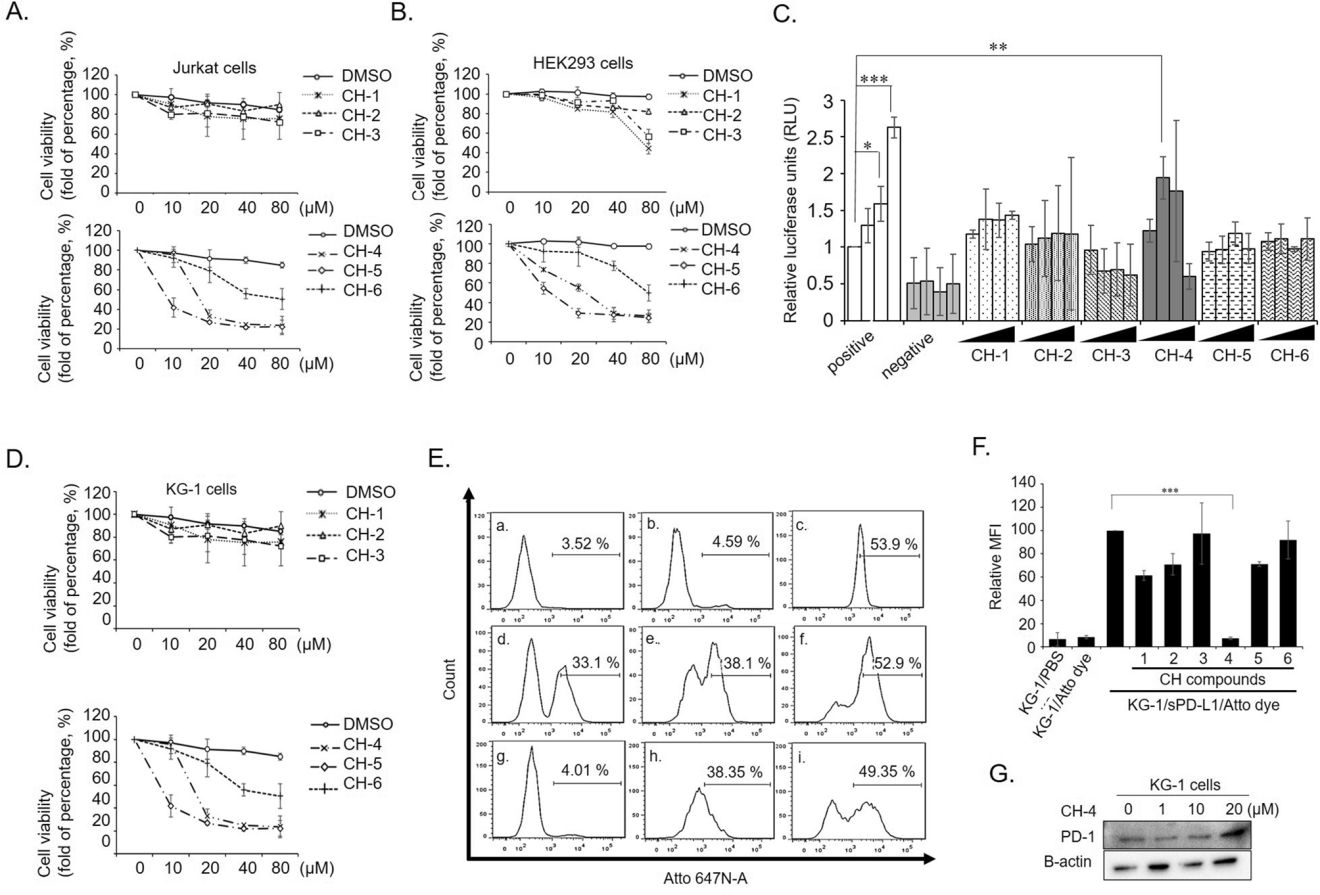
In vitro screening of leading compounds. (**A**) Jurkat cells and (**B**) HEK293 cells were treated with six leading compounds to test cytotoxicity. Cells were seeded into 96-well plates at a density of 7 × 10^3^/well and incubated overnight. The cells were then treated for 48 h with different concentrations (0, 10, 20, 40, and 80 µM) of the six CH compounds. At 48 h, cell cytotoxicity WST-1 assays were performed. The x-axis indicates treatment concentrations, while the y-axis indicates the percentage of cell viability (each absolute absorbance value [abs. 450 nm–630 nm] was normalized with the controls). (**C**) The capacity of the CH compounds to inhibit the PD-1/PD-L1 interaction was tested using the bioluminescent cell-based assay. Effector cells (growth-arrested reporter Jurkat cells) were preincubated with anti-PD-1 neutralizing antibody (Cat No. 71120), DMSO, or each of the CH compounds. The activity of luciferase was determined as an indicator of cell activation. The graphs present mean ± SD (standard deviation) values from at least three independent experiments. (**D**) After overnight incubation, KG-1 cells were treated for 48 h with increasing concentrations of the CH compounds (0, 10, 20, 40, or 80 µM). (**E**) KG-1 cells were incubated with CH compounds prior to sPD-L1-Atto staining. Flow cytometry determined binding of the Ni–NTA-labeled sPD-L1 (PD-L1-Atto) complex to KG-1 cells expressing PD-1. Cell staining (Atto-subset) was blocked in the presence of the CH compounds. The experimental groups are indicated as (a) KG-1/PBS, (b) KG-1/Atto dye, (c) KG-1/sPD-L1/Atto dye, (d) KG-1/sPD-L1/Atto dye/CH-1, (e) KG-1/sPD-L1/Atto dye/CH-2, (f) KG-1/sPD-L1/Atto dye/CH-3, (g) KG-1/sPD-L1/Atto dye/CH-4, (h) KG-1/sPD-L1/Atto dye/CH-5, (i) KG-1/sPD-L1/Atto dye/CH6. (**F**) The cell staining data from (**E**) are normalized and quantified as relative MFI values. (**G**) At 48 h, PD-1 protein (20 µg/well) expression was determined by Western blotting. The bar graphs present the mean ± SD (standard deviation) values from three independent experiments. *p < 0.05, **p < 0.01, ***p < 0.001.

The WST-1 cytotoxicity assay evaluated cytotoxicity in effector cells (Jurkat cells) (Fig. 2A) and target cells (HEK293 cells) (Fig. 2B). The OD value that resulted from WST-1 reagent dye formation strongly correlated with the number of metabolically live cells. To further characterize cytotoxicity tolerance, CC_50_ (cytotoxic concentration 50%) values were calculated and are shown in Table 1. Both cell lines displayed high tolerance (exceeding 20 µM) for five compounds (CH-1, 2, 3, 4, and 6), while CH-5 exhibited high cytotoxicity (Jurkat cells CC_50_ = 10.57 ± 2.67 µM; HEK293 cells CC_50_ = 8.52 ± 1.74 µM). Doses of compounds in the screening assay were based on the CC_50_ values of each cell line. Thus, screening concentrations of CH-1, -2, -3, -4 and -6 were 0, 10, 20 and 40 µM, respectively, while the concentrations for CH-5 were 0, 0.1, 1 and 10 µM, respectively. To ensure reliable screening, anti-PD-1 neutralizing antibody (Cat No. 71120) was used as a positive control for inhibiting the interaction of PD-1/PD-L1, while HEK293 cells transfected with control plasmid served as the negative control. These groups were treated with increasing concentrations of anti-PD-1 neutralizing antibody (0, 0.1, 1 and 10 µg/mL) and luminescence intensity was determined after treatments. In the positive control group, anti-PD-1 antibody exhibited increasingly higher inhibitory qualities with increasing antibody concentration, whereas anti-PD-1 neutralizing antibody had no effect on the negative control. Compared with the control groups, CH-4 showed potential inhibitory capacity for the PD-1/PD-L1 interaction at a dose of 10 µM. The other CH compounds failed to show significant inhibitory capacity in this assay. These data indicated that CH-4 could be a potential SMI for blocking the PD-1/PD-L1 interaction (Fig. 2C).

**Table 1:** CC_50_ values of PD-1 inhibitors tested in Jurkat and 293 cells.

Compounds	Jurkat cells (μm)	293 cells (μm)
CH-1	132.80 ± 5.10	46.89 ± 10.18
CH-2	94.91 ± 1.97	151.10 ± 32.52
CH-3	211.72 ± 46.46	78.13 ± 4.95
CH-4	30.33 ± 1.30	18.87 ± 1.53
CH-5	10.57 ± 2.67	8.52 ± 1.74
CH-6	103.39 ± 0.94	112.91 ± 26.12

The CC_50_ value is presented as the mean ± standard deviation.

Previous investigations into plasma taken from cancer patients have suggested that the soluble form of PD-L1 (sPD-L1) is a negative regulator of T cell activation^31,37^. Using the WST-1 assay, CH compound cytotoxicities were quantified at different concentrations in KG-1 cells (Fig. 2D). CH-1, -2, -3, -4, and -6 showed low cytotoxicity to KG-1 cells at 10 µM, whereas KG-1 cells were sensitized to CH-5. The data indicated an appropriate concentration for testing inhibition of the PD-1/sPD-L1 interaction by flow cytometry. To clarify the inhibitory qualities of CH compounds in sPD-L1/PD-1 interactions, His-tagged sPD-L1 recombinant protein was labeled with Ni–NTA-conjugated fluorescent dye (Atto) and flow cytometry analysis was performed (Fig. 2E). Incubation of KG-1 cells with Ni–NTA-labeled sPD-L1 revealed a clear staining (Fig. 2E [c], KG-1/sPD-L1/Atto dye). After incubating sPD-L1 with Ni–NTA-labeled dye for 1 h, CH-4 treatment induced a slight, yet significant, shift in staining intensity (Fig. 2E [g], KG-1/sPD-L1/Atto dye/CH-4) compared with controls (Fig. 2E [a], KG-1/PBS; [b], KG-1/Atto dye). In contrast, the other compounds failed to inhibit or only slightly inhibited the KG-1 cell/sPD-L1 interaction, as shown by increasing staining intensity (Fig. 2E [d–f, h and i]). The relative percentages of mean fluorescence intensity (MFI) values were normalized and are shown in Fig. 2F. When KG-1 cells were treated with increasing concentrations of CH-4, PD-1 expression was unchanged (Fig. 2G).

### In vitro characterization of the inhibitory capacities of CH-4 analogs

**Figure 3:**
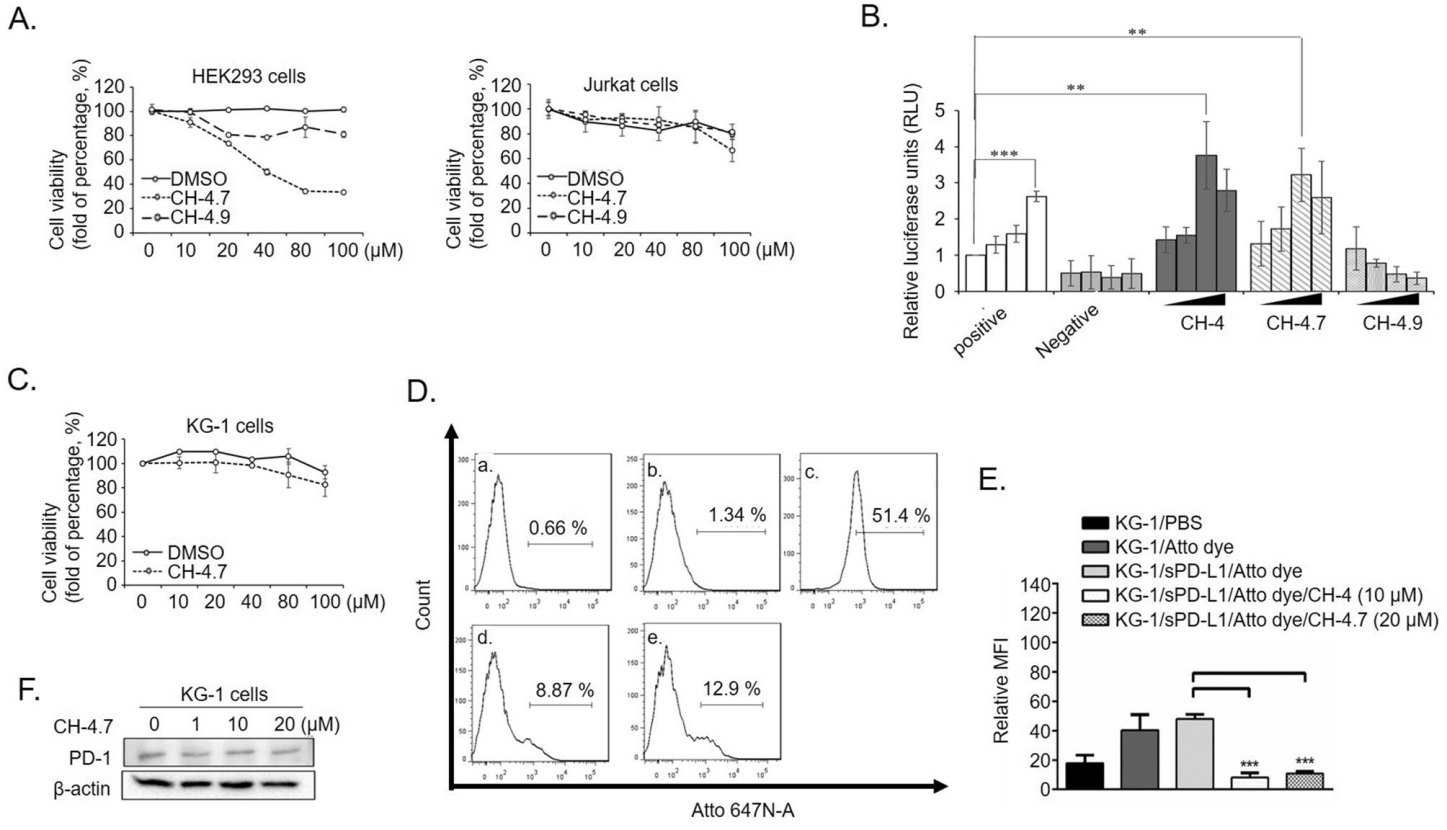
In vitro inhibition by the CH-4 analog of the PD-1/PD-L1 interaction. (**A**) HEK293 cells (left-hand panel) and Jurkat cells (right-hand panel) were treated with CH-4.7 or CH-4.9 to test cytotoxicity. Cells were seeded into 96-well plates at a density of 7 × 10^3^/well and incubated overnight, then treated with increasing concentrations of CH-4.7 or CH-4.9 (0, 10, 20, 40, 80, or 100 µM) for 48 h. At 48 h, cell cytotoxicity WST-1 assays were performed. The x-axis indicates treatment concentrations, while the y-axis indicates the percentage of cell viability (each absolute absorbance value [abs. 450 nm–630 nm] was normalized with the controls). (**B**) The capacities of CH-4.7 and CH-4.9 to inhibit the PD-1/PD-L1 interaction were tested by the flow cytometry assay. The graphs present the mean ± SD (standard deviation) values from at least three independent experiments. (**C**) CH-4.7 cytotoxicity in KG-1 cells. (**D**) Flow cytometry determined binding of the Ni–NTA-l-labeled sPD-L1 (PD-L1-Atto) complex to KG-1 cells expressing PD-1. Cell staining (FITC-subset) was blocked in the presence of CH-4.7. The experimental groups are indicated as (a) KG-1/PBS, (b) KG-1/Atto dye, (c) KG-1/sPD-L1/Atto dye, (d) KG-1/sPD-L1/Atto dye/CH-4 (10 µM), (e) KG-1/sPD-L1/Atto dye/CH-4.7 (20 µM). (**E**) The cell staining data from (**D**) are normalized and quantified as relative MFI values. (**F**) At 48 h, PD-1 protein (20 µg/well) expression was determined by Western blotting. The bar graphs present the mean ± SD (standard deviation) values from three independent experiments. *p < 0.05, **p < 0.01, ***p < 0.001.

To further improve the inhibitory capacity of CH-4, its functional group was replaced by alternative molecules and two CH-4 analogs emerged; CH-4.7 and CH-4.9. In cytotoxicity assays, concentrations exceeding 20 µM for both CH-4.7 and CH-4.9 were associated with increasing cytotoxicity in HEK293 cells (Fig. 3A). In contrast, neither analog showed any evidence of cytotoxicity in Jurkat cells. These data provided appropriate concentrations of CH-4.7 and CH-4.9 in the bioluminescent cell-based screening assay, which examined their inhibitory capacities against the PD-1/PD-L1 interaction. As shown in Fig. 3B, the inhibitory capacity of CH-4.7 was equal to that of CH-4, whereas CH-4.9 failed to inhibit the PD-1/PD-L1 interaction. CH-4.7-induced cytotoxicity was minimal in KG-1 cells (Fig. 3C). After incubating KG-1 cells with CH-4.7, Ni-NTA-labeled sPD-L1 was added to perform an interaction reaction. Compared with the control groups (Fig. 3D [a], KG-1/PBS; [b] KG-1/Atto dye and [c], KG-1/sPD-L1/Atto dye), incubation of CH-4 with the Ni-NTA-labeled sPD-L1 complex significantly decreased the shift in fluorescence intensity (Fig. 3D [d], KG-1/sPD-L1/Atto dye/CH-4). Interestingly, the shift in fluorescence intensity induced by CH-4.7 in the Ni-NTA-labeled sPD-L1/PD-1 complex was similar to that with CH-4 (Fig. 3D [e]). The relative percentage of MFI values was normalized and is shown in Fig. 3E. In dose-dependent investigations, CH-4.7 failed to alter PD-1 protein expression (Fig. 3F).

### CH-4.7 is a potential PD-L1/PD-1 inhibitor for immune checkpoint therapy

**Figure 4:**
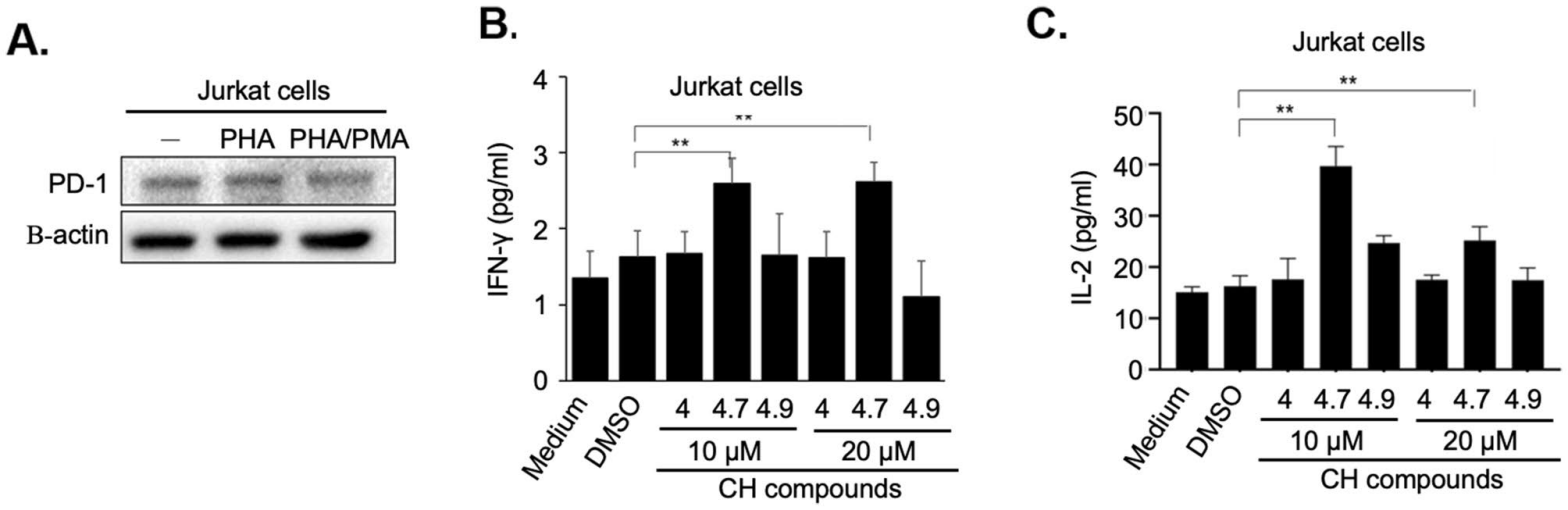
CH-4.7 shows sustained in vitro inhibition of PD-L1/PD-1 and T cell activation. (**A**) Jurkat cells were treated with PHA alone or in combination with PMA for 48 h and PD-1 protein (20 µg/well) expression was determined by Western blotting. Production of IL-2 (**B**) and IFN-γ (**C**) was tested by ELISA. Jurkat cells were incubated with sPD-L1 alone or in combination with CH-4, 4.7 and 4.9 (10 and 20 μM) for 48 h, then analyzed for IL-2 or IFN-γ content. CH-4.9 served as the negative control. The bar graphs present the mean ± SD (standard deviation) values from three independent experiments. **p < 0.01, ***p < 0.001.

Studies have shown that the interaction of PD-L1/2 with PD-1 effectively reduces cytokine production, such as IL-2 and IFN-γ^38–40^. To further characterize the inhibitory effects of CH-4.7 against the PD-L1/PD-1 interaction, we quantified PMA- and PHA-induced production of IL-2 and IFN-γ in activated Jurkat cells. As shown in Fig. 4A, neither PHA nor PMA altered PD-1 protein expression in Jurkat cells. To determine the inhibitory efficiency of CH-4.7, Jurkat cells were co-treated with sPD-L1 and either CH-4.7 (10 µM) or CH-4.9 (20 µM), then incubated with PMA or PHA for 48 h. At the indicated time points, IL-2 and IFN-γ production was analyzed by ELISA (Fig. 4B,C). At the concentration of 10 µM, CH-4.7 effectively inhibited the PD-1/PD-L1 interaction and was associated with higher levels of IL-2 and IFN-γ production compared to outcomes observed with the control group and other CH compounds. Thus, according to measurements of IL-2 and IFN-γ production, CH-4.7 was considered to be an effective inhibitor for blocking the PD-L1/PD-1 interaction.

## Discussion

**Figure 5:**
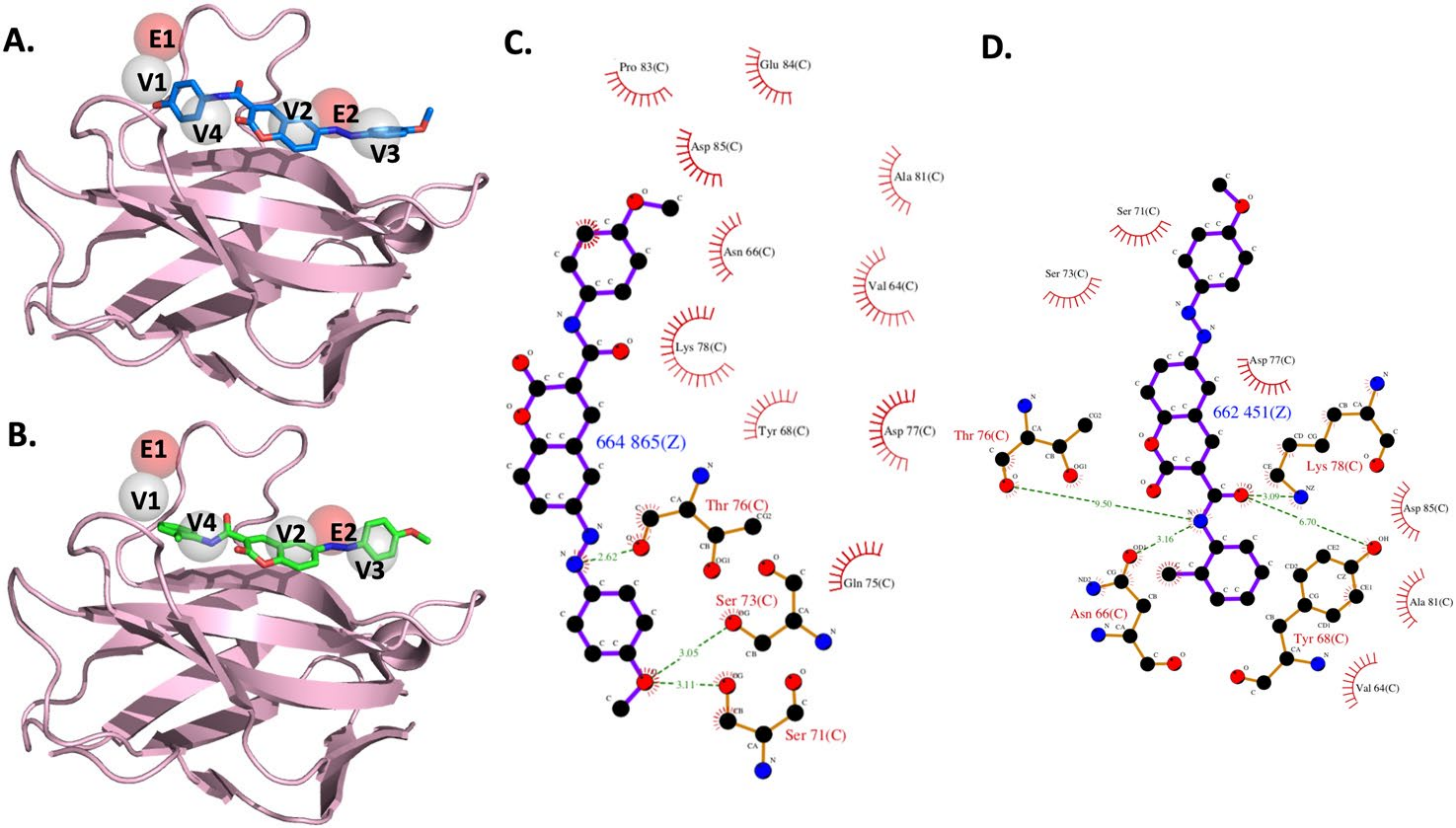
Docked compounds show a high binding affinity towards PD-1. (**A,B**) Visualization of the docked compounds with anchors in the human PD-1 structure, which is illustrated by the pink cartoon. Six anchors are shown as transparent red and gray spheres, while the docked CH-4 and CH-4.7 are shown as blue (**A**) and green (**B**), respectively. (**C,D**) The interaction diagrams between the proteins and docked (**C**) CH-4 or (**D**) CH-4.7 are shown as 2-D plots, which were examined by LIGPLOT^41^.

In this study, we selected the human PD-1 protein structure as the target protein and used the molecule docking method to screen more than 200,000 compounds from the NCI database. The post-virtual screening analysis was processed by the SiMMap server to generate the anchors containing the interaction type, essential binding residues and functional moieties. Based on the SiMMap score, six compounds were obtained from the NCI database and validated by rigorous bioassays. CH-4 was initially identified and served as the lead compound. In a series of experiments involving 11 analogs of CH-4, we found that CH-4.7 inhibited the PD-1/PD-L1 interaction to the same extent as CH-4. CH-4 and CH4.7 docking poses with their respective anchors are illustrated in Fig. 5A,B, while SiMMap scores and docking energies are shown in Table 2. Thus, the van der Waals anchors V2, V3 and V4 are integral to both CH compounds.

**Table 2:** SiMMap scores and docking energies for CH-4 and CH-4.7.

Compounds	Score^+^	Energy^∞^	Jurkat cells CC_50_ (μm)	293 cells CC_50_ (μm)
CH-4	4.364	− 90.265	30.33 ± 1.30	18.87 ± 1.53
CH-4.7	3.360	− 87.577	> 100 μm	40.02 ± 3.71

The CC_50_ value is presented as the mean ± standard deviation.

^+^From SiMMap.

^∞^From iGEMDOCK.

**Figure 6:**
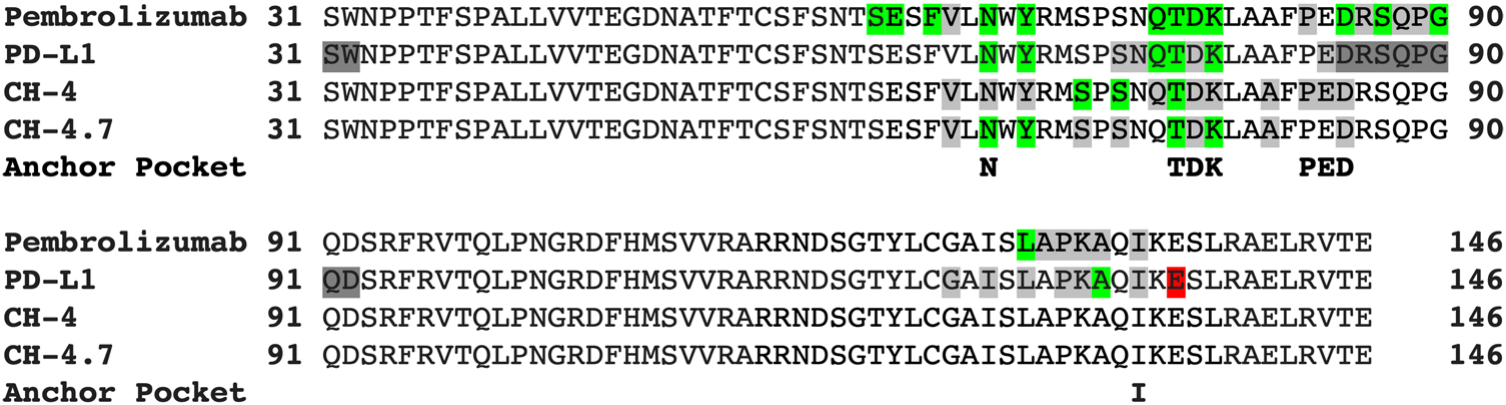
Interaction profiles of PD-1. Interaction profiles were generated by PDBsum^42^ using PD-1 structures complexed with pembrolizumab Fv (PDB ID: 5B8C) and PD-L1 (PDB ID: 4ZQK). LIGPLOT^41^ examined the interaction between PD-1 and CH-4 and also CH-4.7. The interactive resides in PD-1 are highlighted as red for the salt-bridge interaction, green for the H-bonding interaction, and light gray for the non-bonding interaction. The residues highlighted as dark gray are unmodeled residues of the PD-1 domain containing the 4ZQK structure.

The interaction profiles of PD-1 and other molecules, including pembrolizumab Fv, PD-L1, CH-4 and CH-4.7, are illustrated in Fig. 6. We used the protein–protein interaction analysis tool from PDBsum^42^ to examine the interaction residues, using human PD-1 structures complexed with pembrolizumab Fv (PDB ID: 5B8C) and PD-L1 (PDB ID: 4ZQK)^43^. Eight unmodeled residues (31S, 32 W, 85D, 86R, 87S, 88Q, 89P, and 90G) exist in the 4ZQK structure of the PD-1 domain. However, six unmodeled residues (from 85D to 90G) form part of the essential fragment in the interface between PD-1 and PD-L1, which is why we did not select 4ZQK as a target protein structure for virtual screening. The 2D interaction diagrams of CH-4 and CH-4.7 within their pocket environment were examined using the LIGPLOT program^41^ and are shown in Fig. 5C,D. In Fig. 6, the interactive resides in PD-1 are highlighted as red for the salt-bridge interaction, green for the H-bonding interaction, and light gray for the non-bonding interaction. The interactions of these four molecules involve the PD-1 consensus binding residues of N66, Y68, T76, D77 and K78, which are all located in the V2, V3 and V4 anchoring domain. These three anchors may therefore play an essential role in the interference of PD-1 binding with PD-L1.

The US Food and Drug Administration has approved the use of monoclonal antibodies for blocking the PD-L1/PD-1 interaction in various types of cancers^44^. However, while antibody-based immune checkpoint therapies have shown remarkable clinical activity, they are also associated with significant disadvantages, including the fact that the majority of patients fail to respond to treatment, intravenous injections are required, and serious immune-related adverse events have occurred with the loss of immunological self-tolerance^45^. Fortunately, the advent of SMIs offers a potential alternative to monoclonal antibodies, with important clinical advantages that include more efficient uptake by the cell membrane, more specific targeting of diseased organs, prolonged therapeutic effects, lower acquisition costs, and their oral administration, which may be preferred by patients over intravenous or subcutaneous injections of monoclonal antibodies^6^. In this study, cell-based PD-L1/PD-1 interaction screening identified a potential PD-1 SMI (CH-4.7). Its inhibitory capacity was tested by blocking the membranes of Jurkat and KG-1 cells and also the soluble form of PD-L1 that interacts with PD-1 (Figs. 2 and 3). CH-4.7 effectively inhibited the PD-L1/PD-1 interaction and stimulated the production of IL-2 and IFN-γ, without influencing immune cell activity or cytokine secretion (Fig. 4). Cytotoxicity testing demonstrated minimal effects on THP-1 and Jurkat T cell lines. In the cell-based screening assay, CH-4.7 displayed a higher inhibitory capacity than PD-1 antibody treatment (Fig. 3C).

Few evaluations exist of SMIs that target PD-1. Instead, the majority of SMIs that have been developed have focused on PD-L1 or related signaling molecules^46^. PD-L1 is overexpressed on the cell membrane of different types of cancers^1,35,47,48^ and this overexpression enables PD-L1 to bind with PD-1 and suppress T cell-mediated antitumor immune responses, fostering tumor growth^35^. Our study focused on PD-1 SMI development and our evidence showed that CH-4.7 specifically targets PD-1 and sustains T cell activation, as according to IL-2 and IFN-γ production (Figs. 3 and 4). As shown in Fig. 3A, testing of cytotoxicity with CH-4.7 in both Jurkat and HEK293 cells revealed that HEK293 cell viability was reduced to almost 50% at the concentration of 40 μM (Fig. 3A, left panel). In Fig. 3B, CH-4.7 inhibition efficiency was reduced with the higher concentration (40 μM), due to the fact that CH-4.7 had higher cytotoxicity with HEK293. Thus, it is possible that CH-4.7 has superior inhibition efficiency under higher treatment concentrations, but the cell-based screening method used in this investigation could not detect CH-4.7 inhibition efficiency beyond 20 μM.

Crucially, animal experiments are vital in research such as this. Our experiments have yielded reliable in vitro data that give us confidence to test the PD-1 inhibitors in vivo. Testing of several cancer cell lines for cytotoxicity of the PD-1 inhibitors revealed high tolerance among some of those cell lines to the PD-1 inhibitors (data not shown). In addition, testing for cytotoxicity of PD-1 inhibitors with human peripheral blood mononuclear cells (PBMCs) has shown that higher doses of the PD-1 inhibitors exhibit minimal cytotoxicity (data not shown).

In conclusion, we have shown that CH-4.7 effectively inhibits the PD-L1/PD-1 interaction and sustains the activation of cytokines that suppress tumor proliferation. This study provides highly relevant information about the potential therapeutic possibilities of CH-4.7, which deserves to be investigated further and perhaps satisfy unmet medical needs.

## Materials and methods

### Cell lines and reagents

The American Type Culture Collection supplied all cell lines. HEK293 cells were cultured in Dulbecco’s Modified Eagle Medium (DMEM) supplemented with 10% fetal bovine serum (FBS) and 1% penicillin/streptomycin. Jurkat cells were maintained in RPMI 1640 medium supplemented with 10% FBS, 50 uM β-mercaptoethanol and 1% penicillin/streptomycin. KG-1 cells were maintained in Iscove’s Modified Dulbecco Media (IMDM) supplemented with 20% FBS and 1% penicillin/streptomycin. All cells were maintained in a humidified 5% CO_2_ environment at 37 °C. PD-1 antibody was used in this study (Cat No. NBP1-77276, Novus Biologicals, USA).

### Dataset preparation and virtual screening

Human PD-1 (Protein Data Bank [PDB] ID: 5B8C, Chain C) served as the target protein for virtual screening^49^. The structure of 5B8C represents the crystal structure of human PD-1 in complex with pembrolizumab Fv^21^, which was determined by X-ray crystallography at a resolution of 2.15 $\mathrm{\AA }$. The docking pocket of the target protein PD-1 was extracted by collecting the residues around the pembrolizumab 10 $\AA$. We selected 208,023 compounds from the National Cancer Institute (NCI) compound database^50^ for virtual screening and filtered them according to Lipinski’s Rule of Five^51^. After preparing the target protein structure and compound databases, we used the iGemdock program^52^ for virtual screening.

A total of 1,000 top-ranking compounds based on docking energy were submitted to the SiMMap server for re-evaluation^53^, to analyze the conserved interacting residues and specific physicochemical properties of the binding pocket. Binding site properties can be described by the site-moiety map with several anchors, elucidating the interaction between target proteins and docked compounds. Each anchor consists of a binding pocket with corresponding interacting residues, moiety preference, and interaction type (E: electrostatic, H: hydrogen-bonding, or V: van der Waals force). Identified compounds were reordered with the SiMMap score. We then selected potential PD-1 inhibitors (CH compounds) based on their ranking and requested them from the NCI for closer inspection.

### Cell cytotoxicity assay

The WST-1 assay assessed cell cytotoxicity. Briefly, cells were seeded in 96-well plates at a density of 10^3^ cells per well (100 μL/well) with 10% FBS in the medium and incubated for 48 h, before undergoing a further 1 h of incubation at 37 °C with 10 μL of WST-1 solution added to each well**.** Cell viability was quantified by colormetric detection using an ELISA plate reader (Beckman Coulter PARADIGM Detection Platform; Beckman Coulter, IL, USA) at absorbance values of 450 nm and 690 nm to generate an optical density proportional to the relative abundance of live cells in the wells.

### Western blotting

Cells were washed with 1X phosphate-buffered saline (PBS) and harvested by scraping with RIPA buffer (100 mM Tris, 5 mM EDTA, 5% NP40; pH8.0) and proteinase inhibitors (1 mM phenylmethylsulphonyl fluoride, 1 μg/mL aprotinin, 1 μg/mL leupeptin). Proteins (40 µg) were separated on 8% SDS-PAGE gel before being transferred onto polyvinylidene fluoride (PVDF) membranes. Blots were blocked in 5% skimmed milk in 1X PBS for 1 h at room temperature then incubated overnight with actin, PD-1 or PD-L1 primary antibody (1:1,000) at 4 °C. After undergoing washing using 1X PBS, the membranes were incubated with HRP-conjugated secondary antibodies (1:5,000) and visualized using the Bio-Rad enhanced chemiluminescence (ECL) imaging system.

### Cell-based PD-1/PD-L1 checkpoint assay

One day before transfection, HEK293 cells were seeded at a density of 35,000 cells per well in 100 μL of growth medium, to ensure 90% confluence at the time of transfection. The next day, 1 μg of expression vectors of a T cell receptor (TCR) activator and human PD-L1 were transfected into the cells, following the manufacturer’s protocol**.** One day after transfection, a vial of the growth-arrested effector cells (Jurkat cells) stored in liquid nitrogen was quickly thawed in a 37 °C water bath, then transferred to a tube containing 10 mL of assay medium. After spinning the cells at 1,500 revolutions per minute (rpm), the supernatant was removed and the cells were resuspended in 7 mL of prewarmed assay medium. To test the PD-1 inhibitors, the PD-1 antibody was subjected to serial dilutions in assay medium and served as the positive control (2X dilution of antibody was used as the working concentration). The growth-arrested effector cells were preincubated with the diluted PD-1 inhibitors for 15–30 min, then added with the inhibitors to the gene-manipulated HEK293 cells (50 μL/well). Finally, 50 μL of growth-arrested effector cells was added to the HEK293 cells.

Effector cells incubated with anti-PD-1 neutralizing antibody and target cells (PD-L1/TCR-overexpressed HEK293 cells) represent the positive control. Effector cells incubated with anti-PD-1 neutralizing antibody and target cells (only TCR-overexpressed HEK293 cells) served as negative controls. The concentrations of the CH compounds used in this assay were 0, 10, 20, and 40 µM. After ~ 16 h, we performed the One-Step Luciferase Assay System. Briefly, we thawed Luciferase Reagent Buffer (Component A) at room temperature and mixed it well before use. Immediately prior to performing the luciferase assay, we prepared the luciferase assay working solution by diluting Luciferase Reagent Substrate (Component B) with Component A at a 1:100 ratio and mixed it well. We then added 100 μL of One-Step Luciferase Reagent to each well and rocked them at room temperature for ~ 30 min. Luminescence was determined by a luminometer. The average background luminescence (cell-free control wells) was subtracted from the luminescence readings of all wells.

### PD-1/sPD-L1 checkpoint assay

The effect cells were stimulated with anti-CD3 antibody in the presence of recombinant human sPD-L1^31^. This involved coating the 96-well white flat-bottom plates with 5 μg/mL of anti-CD3 antibody or isotype control solution in PBS. After incubating the plates overnight at 4 °C, the antibody solution was removed and the plates were washed 3 times with PBS then dried. sPD-L1 (amino acids [aa] 18–134) was diluted by PBS supplemented with penicillin/streptomycin (each at a final concentration of 100 U/mL) and incubated with each CH compound. The dimethyl sulfoxide (DMSO) was used as the control group. A total of 15 μL of the solution was then added to each well of the antibody-coated plate. Effect cells were centrifuged and diluted to 50,000 per mL, before adding 60 μL of the cell solution to each well. The final concentration of sPD-L1 was 10 μg/mL (0.6 μM). The final concentrations of the CH compounds were 0, 1, 10 and 20 μM, with sPD-L1 molar ratios of 1:5, 1:2, 2:1 and 5:1, respectively. The cells were then cultured for 24 h and luciferase activity was determined by the Bio-Glo Luciferase Assay System (Promega), according to the manufacturer’s instructions.

### Flow cytometry measurements

Binding of sPD-L1 (aa 18–134) to KG-1 T cells was evaluated by flow cytometry, with minor modifications^31^. Briefly, His-tagged PD-L1 protein was stained with Ni-NTA-Atto 647 N fluorescent dye (PD-L1-Atto) (Sigma Aldrich) for 2 h at 22 °C, with an 8:1 molar ratio (protein:dye). PD-L1-Atto was formulated in 150 μL PBS with the tested compounds. The KG-1 cells were incubated with CH compounds for 30 min at 4 °C in darkness. The cells were then centrifuged, washed with PBS and suspended in fresh PBS at a concentration of 1 × 10^6^ cells/mL. sPD-L1-Atto dye was added to each KG-1 cell/CH compound mixture and incubated on ice for an additional 60 min. The final CH compound concentrations were 10 µM. The samples were analyzed using the BD FACS Verse flow cytometer and Flowjo software.

### Cytokine secretion and detection

Jurkat T cells were stimulated with 1 μg/mL PHA (phytohemagglutinin) and 50 ng/mL PMA (phorbol-12-myristate-13-acetate) and then were treated with CH compounds at the concentrations of 10 μM and 20 μM. sPD-L1 was incubated with Jurkat T cells for 48 h, then IL-2 and IFN-γ secretion in the harvested medium was measured with a Human IL-2 and IFN-γ ELISA High Sensitivity kit (Abcam), following the manufacturer’s protocol. Briefly, plates were incubated with biotinylated anti-IL-2 and streptavidin-HRP. Chromogen TMB (3,3',5,5'-tetramethylbenzidine) substrate was added under darkness to each well. After applying the Stop Reagent reaction, the optical density was measured at 450 nm with the reference wave length at 620 nm, using the microplate reader Multiskan Go (Thermo).

### Illustration of 3-D protein structure

PyMOL software was used to draw all of the 3-D figures^54^.

### Statistical analysis

Data were compared between groups using the Student’s *t*-test. All experiments were repeated at least three times, and a *P *value of < 0.05 was considered to indicate statistical significance.

## Supplementary Information

Supplementary Figures.

Supplementary Tables.

## Data Availability

The datasets used and/or analyzed during the current study are available from the corresponding author on reasonable request.

## Abbreviations

DMEM: Dulbecco’s Modified Eagle Medium
ECL: Enhanced chemiluminescence
FBS: Fetal bovine serum
IFN-γ: Interferon gamma
IL-2: Interleukin-2
IMDM: Iscove’s Modified Dulbecco Media
mABs: Monoclonal antibodies
NCI: National cancer institute
NFAT: Nuclear factor of activated T-cells
PBS: Phosphate-buffered saline
PD-1: Programmed cell death protein 1
PD-L1: Programmed cell death ligand 1
PHA: Phytohemagglutinin
PMA: Phorbol-12-myristate-13-acetate
PVDF: Polyvinylidene fluoride
sPD-L1: Soluble form PD-L1
SMIs: Small molecule inhibitors
TCR: T cell receptor
TMB: 3,3′,5,5′-Tetramethylbenzidine
