# Supplementary figures and images for: In vitro characterization of a small molecule PD-1 inhibitor that targets the PD-l/PD-L1 interaction
Source: Sci Rep. 2022 Jan 7;12:303. doi: 10.1038/s41598-021-03590-4 (PMC8741796; doi:10.1038/s41598-021-03590-4)

Figure 2. G

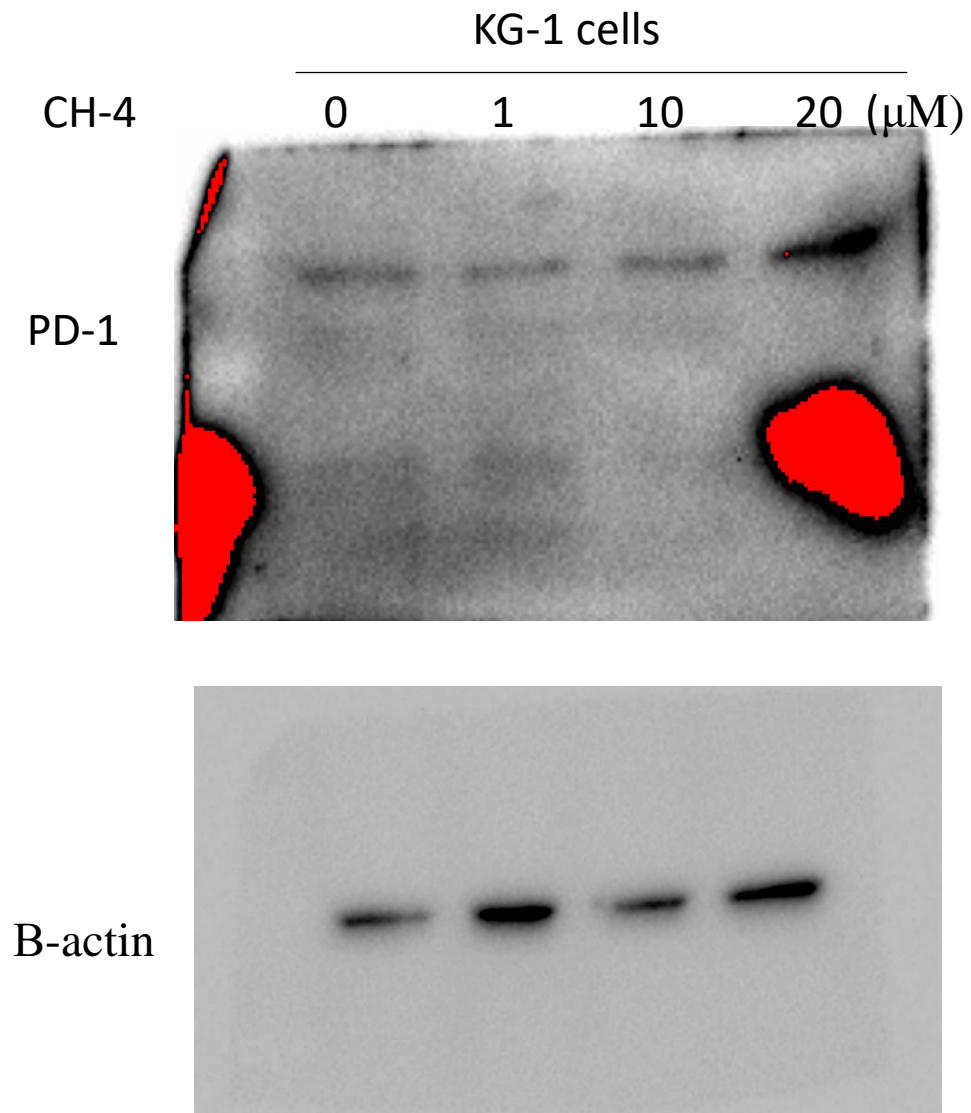

Figure 3. F

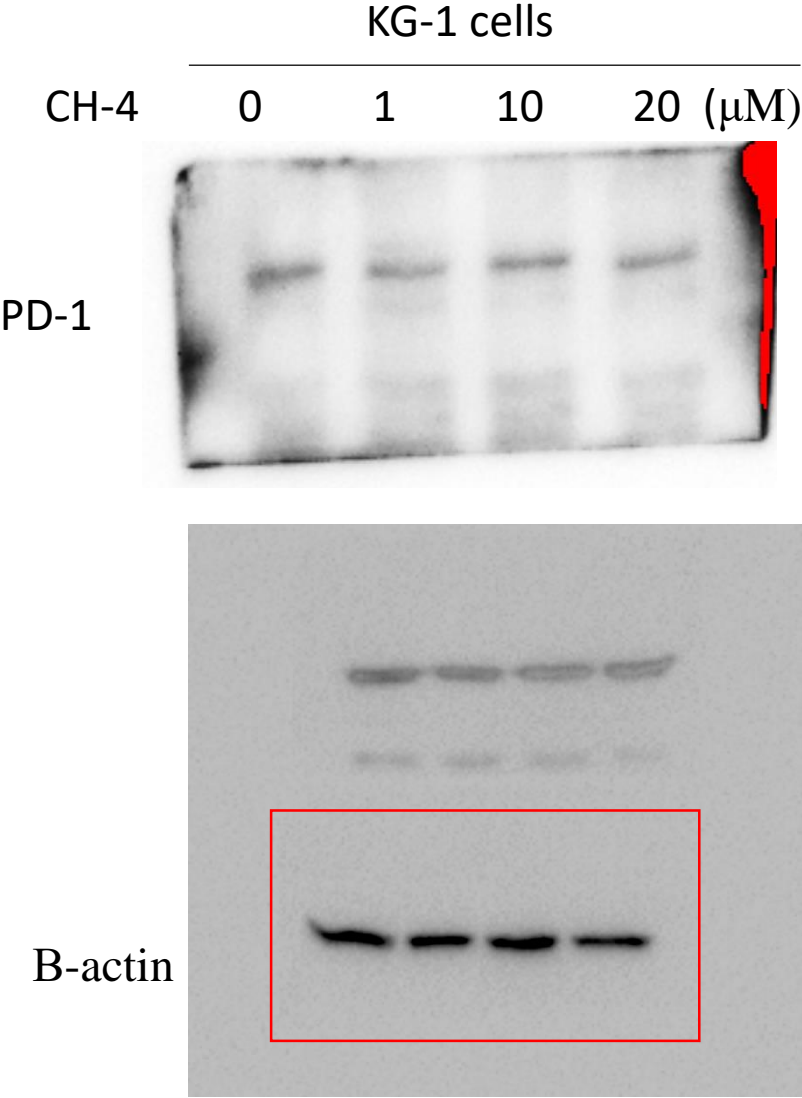

Figure 4. A

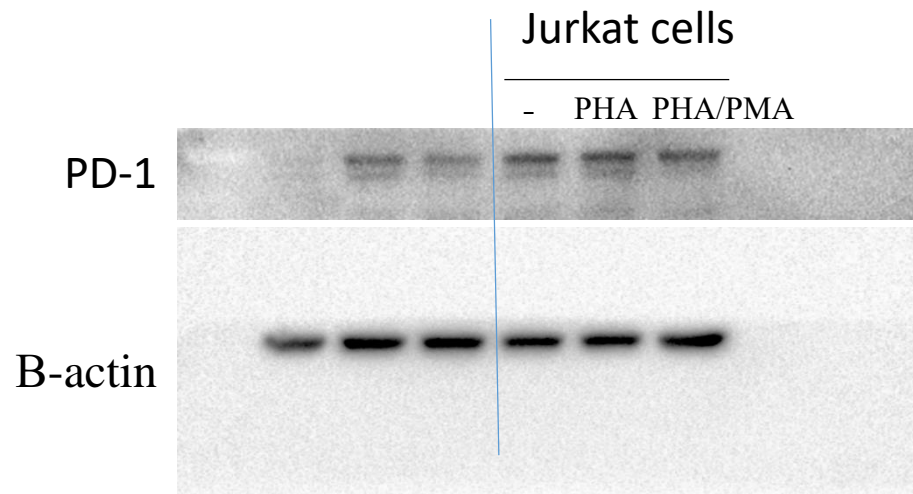

Supplement: Supplementary file 1 — Supplementary Figures. [file 41598_2021_3590_MOESM1_ESM.pdf]
