# Supplementary material for: In vitro characterization of a small molecule PD-1 inhibitor that targets the PD-l/PD-L1 interaction
Source: Sci Rep. 2022 Jan 7;12:303. doi: 10.1038/s41598-021-03590-4 (PMC8741796; doi:10.1038/s41598-021-03590-4)
Supplement: Supplementary file 2 — Supplementary Tables. [file 41598_2021_3590_MOESM2_ESM.pdf]

Supplementary data

Table S1. The top 20 compounds ranked by SiMMap scores identified from virtual screening.

Rank	Compound	S-Score	E1	E2	V1	V2	V3	V4	Available	2-D Structure
1	NSC657666 (CH-1)	5.330	1	0	1	1	1	1	V	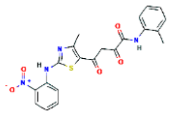
2	NSC715975	4.426	0	0	1	1	1	1		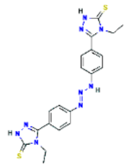
3	NSC65374 (CH-2)	4.422	0	0	1	1	1	1	V	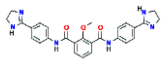
4	NSC68132	4.409	0	0	1	1	1	1		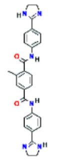
5	NSC88647	4.409	0	0	1	1	1	1		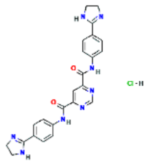
6	NSC49437 (CH-3)	4.405	0	0	1	1	1	1	V	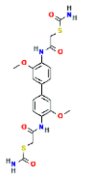
7	NSC73467	4.393	0	0	1	1	1	1		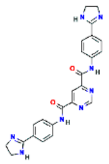
8	NSC625828	4.377	0	0	1	1	1	1		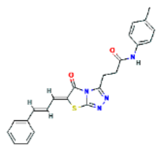

9	NSC29868 (CH-6)	4.369	0	0	1	1	1	1	V	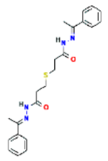
10	NSC631732	4.369	0	0	1	1	1	1		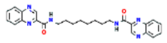
11	NSC664865 (CH-4)	4.364	0	0	1	1	1	1	V	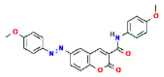
12	NSC45940	4.360	0	0	1	1	1	1		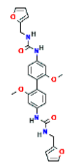
13	NSC521576	4.359	0	0	1	1	1	1		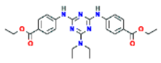
14	NSC730245	4.358	0	0	1	1	1	1		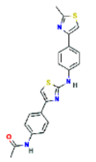
15	NSC714619	4.349	0	0	1	1	1	1		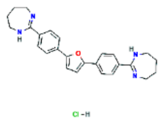
16	NSC59503	4.338	0	0	1	1	1	1		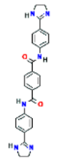
17	NSC718657	4.329	0	0	1	1	1	1		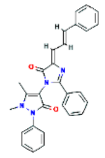

18	NSC710592 (CH-1)	4.325	0	0	1	1	1	1	V	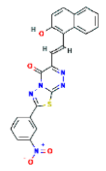
19	NSC521239	4.290	0	0	1	1	1	1		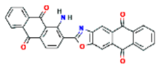
20	NSC282028	3.460	0	0	1	1	0	1		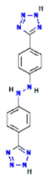

E1, E2, V1, V2, V3 and V4 represent different anchors according to their force types. 1: Part of the compound is located in this anchor. 0: No part of the compound is situated in this anchor.

Abbreviations: S-Rank, SiMMap rank; S-Score, SiMMap score; E1 and E2, electrostatic force; V1-V4, van der Waals force.

Table S2. The 11 CH-4 analogs.

ID	Compound	2-D Structure	ID	Compound	2-D Structure
CH-4.1	NSC641139	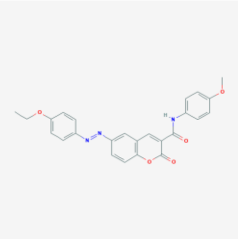	CH-4.7	NSC662451	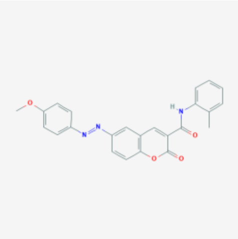
CH-4.2	NSC630069	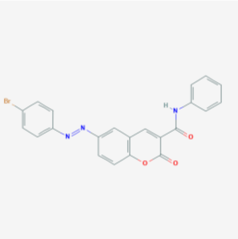	CH-4.8	NSC630075	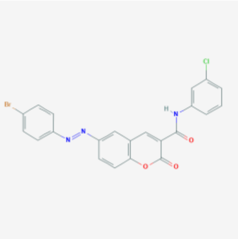
CH-4.3	NSC630071	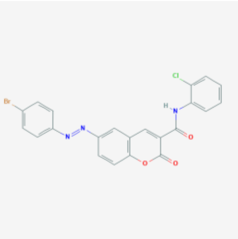	CH-4.9	NSC375104	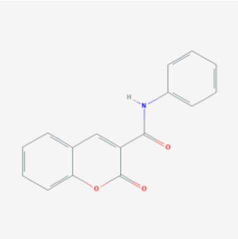
CH-4.4	NSC630072	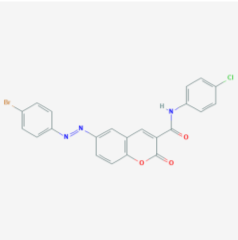	CH-4.10	NSC643596	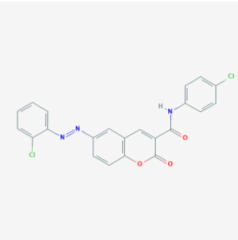
CH-4.5	NSC662450	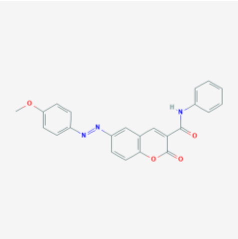	CH-4.11	NSC630074	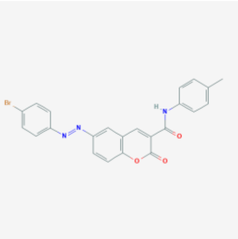
CH-4.6	NSC662452	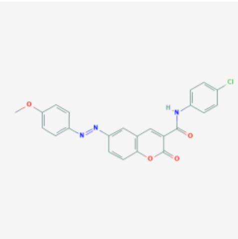			
